# Investigation of the effects of pomegranate juice addition on physicochemical, microbiological, and functional properties of set and stirred yogurts

**DOI:** 10.1002/fsn3.2615

**Published:** 2021-09-29

**Authors:** Mohammad‐Taghi Golmakani, Mohammad Hadi Eskandari, Somayyeh Kooshesh, Mahboobeh Pishan

**Affiliations:** ^1^ Department of Food Science and Technology School of Agriculture Shiraz University Shiraz Iran

**Keywords:** anthocyanin, bioactives, functional yogurt, phenolic compounds, pomegranate juice

## Abstract

Pomegranate juice (PJ) (at concentrations of 13% and 17%) was added to yogurt and its physicochemical and microbial properties were investigated. PJ improved several features of yogurt, bringing an increase in total phenolic contents by 4.3–6.1 and 5.3–7.3 fold in response to 13% and 17% PJ, respectively. Also, there were increases in the total anthocyanin contents of yogurt by 2650–2870 and 3470–3820 fold in response to the said juice concentrations. These increases were observed in both set and stirred yogurts, whereas IC_50_ values of the yogurts decreased by 2.2–2.6 and 3.0–3.3 fold, respectively, compared to the control samples. Total acidity, syneresis, and redness value of the yogurts increased, parallel to the increase in the PJ concentration being added. Also, *Streptococcus thermophilus* count decreased significantly, whereas no significant effect was observed on the population count of *Lactobacillus delbrueckii* subsp. *bulgaricus*. Among PJ yogurt samples, the panelists selected the 13% PJ stirred yogurt as the best sample. PJ was observed to contain valuable bioactive compounds with functional and medicinal effects that culminate in health benefits.

## Graphical abstract

The addition of bioactive compounds, such as polyphenols, flavonoids, and anthocyanins, to the yogurt can make it healthier. Pomegranate juice is a valuable source of natural antioxidants, especially for its phenolic compounds, and can be added to the yogurt for improving its functional properties and to fulfill consumer expectations. The objective of this study was to optimize pomegranate juice concentrations (13% and 17%) by adding them to the yogurt, followed by an analysis of its physicochemical features and microbial properties.

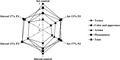

## 1 INTRODUCTION

Pomegranate (*Punica granatum* L.) belongs to the family Punicaceae and is planted around the world in various microclimatic areas. Iran, USA, Turkey, Egypt, Italy, India, Chile, and Spain are the leading countries in pomegranate production (Farahmand et al., 2017). Annually about 3 million tons of pomegranates are produced in the world, among which Iran is the largest producer with an annual production of 940,000 tons (Troujeni et al., 2018). Pomegranate contains valuable bioactive compounds such as polyphenols, flavonoids, and anthocyanins in its various parts, with functional and medicinal properties, i.e., antioxidant activities, anticancer benefits, and anti‐atherosclerotic actions. Pomegranate juice (PJ) is a nutritious drink and is consumed frequently for its sweet and tart taste which results from the concomitant presence of glucose and phenolic compounds in the fruit. Its phenolic compounds are anthocyanins, ellagic acid, phytoestrogenic flavonoids, and tannins (Farahmand et al., 2017).

Yogurt is a popular fermented dairy product and is commonly consumed for its health benefits and nutritional properties. Beneficial effects of yogurt have been associated with the presence of calcium; phosphorus; potassium; vitamins A, B_2_, and B_12_; high biological value proteins; and essential fatty acids. Yogurt is also a well‐known probiotic carrier, whereas *Bifidobacterium*‐ and *Lactobacillus*‐enriched yogurts are among the most common types of functional foods. Consumption of yogurt has been linked with significant health benefits, including prevention of osteoporosis, diabetes, and cardiovascular diseases, while promoting general gut health and modulating the immune system (Hadjimbei et al., 2020). The consumption of yogurt is increasing rapidly in different parts of the world and, in recent decades, yogurt recipes have diversified as a result of creativity by producers and in response to the consumers' demands for healthier and tastier products, which has collectively led to the development of a range of products with different flavors, consistencies, and textures (Morell et al., 2015). In particular, the inclusion of fruits in yogurt recipes is gaining popularity, domestically and industrially (Ni et al., 2018). Meanwhile, the addition of fruits or fruit extracts to the yogurt can have major impacts on its physicochemical and nutritional properties (Oliveira et al., 2015). This effect is fruit‐specific and relates to the nutrients and non‐nutrients composition of the fruit. For instance, as a source of phenolic compounds, berries are commonly added to yogurts (Mattila et al., 2006). Phenolic compounds are known to interact with milk proteins and form protein‐polyphenol complexes, as a result of which the nutritional properties of dairy products can be enhanced. Thus, adding PJ to yogurt can provide additional health properties, particularly antioxidant properties and phenolic compounds. Also, this supplementation could lead to development of new functional dairy products that could fulfill consumer demands (Axten et al., 2008). Nguyen and Hwang (2016) reported that total phenolic content (TPC) and total flavonoid content increased proportionally by increasing the aronia juice concentration. The antioxidant activity of yogurts with aronia juice was significantly higher than that of the control, and increased proportionally parallel to the increase in the aronia juice concentration. Jaster et al. (2018) observed that the incorporation of cryoconcentrated strawberry pulp into the yogurt resulted in a 3‐fold increase in total anthocyanin content (TAC) and antioxidant activity. Cușmenco and Bulgaru (2020) noticed that the aronia yogurt had the highest TCP, TAC, and antioxidant activity in comparison with other fruit yogurts. Peach and apple yogurts recorded the same values for TPC and TAC. Moreover, Nign et al. (2021) investigated the supplementation of set yogurt with passion fruit juice and reported that the passion fruit juice increased the TPC and significantly enhanced the antioxidant activity. On the other hand, it showed superior quality in comparison with the corresponding control yogurt.

With a view on the available literature, no work has evaluated the effects of PJ addition on improving the functional properties of set and stirred yogurts. So, the main objective of this study was to determine the effects of different concentrations of PJ on bioactive compounds and antioxidant activity of yogurt samples, while evaluating the quality and sensory characteristics of set and stirred yogurts which resulted from their mixing with PJ.

## 2 MATERIALS AND METHODS

### 2.1 Preparation of PJ

Mature pomegranate fruits (*Punica granatum* cv. Rabab), with no visible external cuts or spoilage, were purchased from a local market in Shiraz, Iran. The pomegranate fruits were peeled manually and the arils were squashed in a blender (Ju2000 Vitae Moulinex, Barcelona, Spain). The resultant PJ was filtered through a cotton gauze to remove fruit debris and unwanted particles. Then, the fruits were stored at a frozen temperature (−18°C) until further analysis (Trigueros et al., 2014).

### 2.2 Chemical materials and starters

Folin‐Ciocalteu reagent and 2,2‐diphenyl‐1‐picrylhydrazyl radical (DPPH°) were supplied by Sigma‐Aldrich. All other chemicals (analytical grade) and culture media were purchased from Merck. Milk powder was purchased from Pegah‐e‐Fars Dairy Company. YF‐L811 yogurt culture was obtained from Chr. Hansen Ltd. and directly used as starter culture.

### 2.3 Yogurt preparation

According to our preliminary experiments (data not shown), the effects of different PJ concentrations were evaluated on primary texture, color, and taste of yogurts. Finally, 13% and 17% PJ concentrations were selected as optimum concentrations. Reconstituted milk was produced at 40 ± 1°C by a moderate mixing of milk powder with distilled water. The reconstituted milk contained 18.48% total solid and 3.03% fat. The milk dispersion was refrigerated at 4°C for 12 h to allow full hydration of the milk powder before usage. Reconstituted milk was pasteurized (85 ± 1°C for 30 min) (Ramirez‐Santiago et al., 2010), cooled (44 ± 1°C), and inoculated with 0.03 g/L freeze‐dried starter cultures (*Streptococcus thermophilus* and *Lactobacillus delbrueckii* subsp. *Bulgaricus*; each count was ~10^10^ CFU/g). Then, PJ was added at 13% and 17% to the yogurts. Regarding the set yogurt, milk fermentation was carried out at 44 ± 1°C until a pH of 4.5–4.6 was achieved. The fermented milk batches were cooled and stored at 4°C for 24 h. For stirred yogurt, after fermentation, the fermented milk batches were cooled and stored at 4°C for 24 h. The obtained gels were removed from the refrigerator and gently stirred by a mechanical mixer (Caframo, RZR1, Cole‐Parmer) at 500 rpm for 1 min. Finally, 13% and 17% concentrations of PJ were added. Both stirred and set yogurts were stored at 4°C until further analysis. Physicochemical examinations were carried out after 1, 5, 15, and 20 days of refrigerated storage (4°C).

### 2.4 Physicochemical properties

#### 2.4.1 Acidity and pH

The pH was determined using a TPS digital pH meter (Ohaus) that was calibrated using pH 4.0 as reference along with 7.0 buffer solutions. The yogurt samples were blended before pH measurement. Also, total titratable acidity was determined by titration with 0.1 N NaOH until the pH value reached 8.1 and the amount of acidity was expressed as the percentage of lactic acid.

#### 2.4.2 Syneresis

Syneresis was analyzed during the storage period by a method proposed by Wang et al. (2020). Forty grams of each sample (4°C) were placed in a tube and centrifuged at 222 *g* for 10 min at 4°C. The clear supernatant was poured off, weighed, and finally expressed as the weight (%) relative to the original weight of the yogurt.

#### 2.4.3 Color attributes

Color measurements were carried out using a HunterLab apparatus (Chroma meter CR‐400/410; Konica Minolta). The color values were expressed as L* (Lightness/darkness), a* (redness/greenness), and b* (yellowness/blueness).

### 2.5 Bioactive compounds

#### 2.5.1 Total phenolic content

Total phenolic content was measured according to the Folin‐Ciocalteu colorimetric method. For this purpose, 0.1 ml of each sample was mixed with 0.75 ml Folin‐Ciocalteu reagent which had been previously diluted 10 times in the presence of 0.75 ml sodium carbonate solution (2%). After being incubated at room temperature for 60 min, the absorbance was measured at 765 nm. Gallic acid was used at concentrations of 50, 100, 500, and 1000 mg/ml, as a measure of the standard curve for phenolic compounds (Farahmand et al., 2017).

#### 2.5.2 Total anthocyanin content

Total anthocyanin content was quantified by a pH differential method and calculated according to equations (1) and (2) (Farahmand et al., 2017). The absorbance was measured at 510 and 700 nm in buffers at pH values of 1.0 and 4.5.

(1) Totalanthocyanincontent(mg/L)=(A×Mw×DF)×1000/(MA×D),

(2) $$A=\left[\left(A_{510}‐A_{700}\right)\text{pH}\;1.0‐\left(A_{510}‐A_{700}\right)\text{pH}\;4.5\right],$$

where Mw (molecular weight) is 449.2 g/mol for cyanidin‐3glucoside (cyd‐3‐glu), DF is the dilution factor, MA is the molar absorptivity (26,900), and *D* is the path length (1 cm).

### 2.6 Radical scavenging activity

Radical scavenging activity (RSA) was measured using DPPH°, as described by Farahmand et al. (2017). A volume of 1 ml of each yogurt sample was added to 19 ml of 0.1 mM methanol solution of DPPH°. The mixture was shaken vigorously and left to stand at room temperature for 60 min in the dark. Then, the absorbance value was recorded at 517 nm against blank. The inhibition percentage of DPPH° was determined according to equation (3):

(3) $$\text{Inhibition}\left(\%\right)=\left(\left(A_{c}‐A_{s}\right)/A_{c}\right)\times 100,$$

where *A*_c_ is absorbance of the control reaction (with all reagents except the test sample) and *A*_s_ is the absorbance of the sample after 60 min.

The sample that culminated in 50% inhibition (IC_50_) was determined from the equation and then obtained from the plotted inhibition curve.

### 2.7 Microbial analysis

An aliquot of 10 g was sampled from each yogurt and was mixed with 90 ml of sterile saline solution (0.85%) in a Stomacher bag. The suspension was homogenized for 1 min with a Stomacher Lab‐Blender (Smasher; AES Chemunex). Serial dilutions were prepared using the same saline solution. *S*. *thermophilus* colonies were counted on M17 agar plates and were incubated under aerobic conditions at 37°C for 48 h, whereas *L*. *delbrueckii* colonies were counted on MRS agar plates and incubated anaerobically for 72 h at 37°C. The bacterial populations of the yogurt samples and controls were counted after 5, 10, 15, and 20 days of storage. The values were expressed as log cfu/ml of the yogurt medium (Demirkol & Tarakci, 2018).

### 2.8 Sensorial evaluation

Sensorial evaluation form was developed according to Arslaner et al. (2021) with some modifications. Sensory descriptors were scored by 20 trained panelists (10 females and 10 males; age range of 20–31 years) from Pegah‐e‐Fars Dairy Company, whereby 5 points of the hedonic scale were used. In the scale, 0 indicated the most unfavorable and 5 indicated the most favorable. Panelists were asked to score the appearance, color, odor, texture, taste, and pleasantness of the yogurt samples.

### 2.9 Statistical analysis

All experiments were performed in triplicate and the data were reported as mean values of the measurements. The data were analyzed by full factorial design using SAS software (version 9.1; SAS Institute Inc.) for comparison among the mean values using Duncan's multiple range test (*p* < .05). Standard deviation values are presented in the tables and the standard deviation bars are provided in the figures.

## 3 RESULTS AND DISCUSSION

### 3.1 Physicochemical properties

#### 3.1.1 Acidity and pH

**FIGURE 1:**
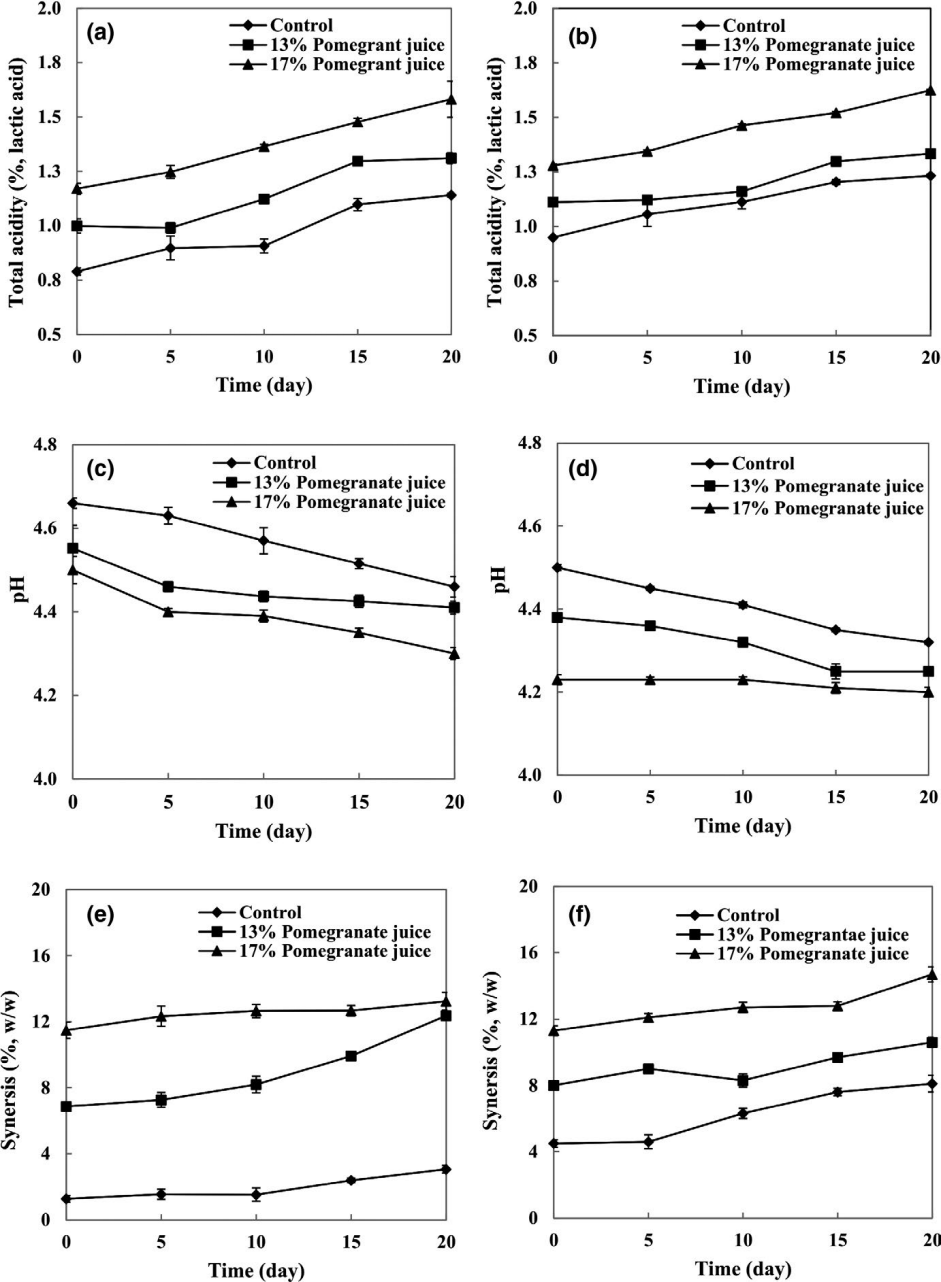
Changes in total acidity, pH, and syneresis of set (a, c, e) and stirred yogurts (b, d, f) containing different concentrations of pomegranate juice during the storage period

Figure 1a,b show the total titratable acidity of set and stirred yogurts, respectively. Total titratable acidity of both set and stirred yogurts increased in response to the increase in PJ concentrations from 13% to 17% according to the high acidity of PJ (1.36%). Similarly, Nguyen and Hwang (2016) reported that the total titratable acidity of yogurt increased in response to an increase in aronia juice concentration. There were increases in the total titratable acidity of the control group and of both set and stirred samples at different concentrations of PJ (13% and 17%) during the storage period.

The pH values of set and stirred yogurts are presented in Figure 1c,d, respectively. The initial pH values of all samples ranged from 4.25 to 4.65. Due to the high acidity of PJ (pH 3.16), increasing its concentration led to a decrease in the pH of PJ‐yogurt samples. Similarly, Jaster et al. (2018) reported that the yogurt acidified with the cryoconcentrated strawberry, meaning a consequent reduction of pH. Also, pH content of strawberry juice put great effects on the strawberry juice yogurt samples (Rahman et al., 2020). The initial pH values of set and stirred yogurts supplemented with 13% PJ were 4.55 and 4.38, respectively, and those supplemented with 17% PJ were 4.50 and 4.23, respectively. In the first concentration, the pH values ultimately reduced to 4.40 and 4.25, respectively. In the second concentration, however, the values reduced to 4.30 and 4.20, respectively, at the end of the storage period. Significant differences were observed between pH values of the stirred and set yogurts, which can be related to their different production processes, especially regarding the step in which PJ was added.

#### 3.1.2 Syneresis

Figure 1e,f show the syneresis of yogurt samples containing different concentrations of PJ during the storage period. Syneresis increased parallel to the increase in PJ concentration, which can be attributed to the higher amount of total titratable acidity and lower pH levels of the PJ‐yogurt samples, as these factors stimulate syneresis in the yogurt (Tamime & Robinson, 1999); since fruit acidity causes reduction in the water binding capacity of proteins, an increase in syneresis is common in fruit yogurts (Roy et al., 2019). Also, syneresis significantly increased during the storage period, and this can be related to the disorganized rearrangement of the casein network (van Vliet et al., 1997). Syneresis was found to be significantly lower in the set yogurts than in the stirred yogurts, probably due to the disruption of the gel network when stirring was done on the PJ‐yogurts to make them stirred yogurts (Ranadheera et al., 2012). At the end of the storage period (20 days), stirred yogurts containing 17% PJ showed the highest syneresis value (13.73%), whereas the control group of set yogurt samples showed the lowest syneresis value (3.69%).

#### 3.1.3 Color attributes

**FIGURE 2:**
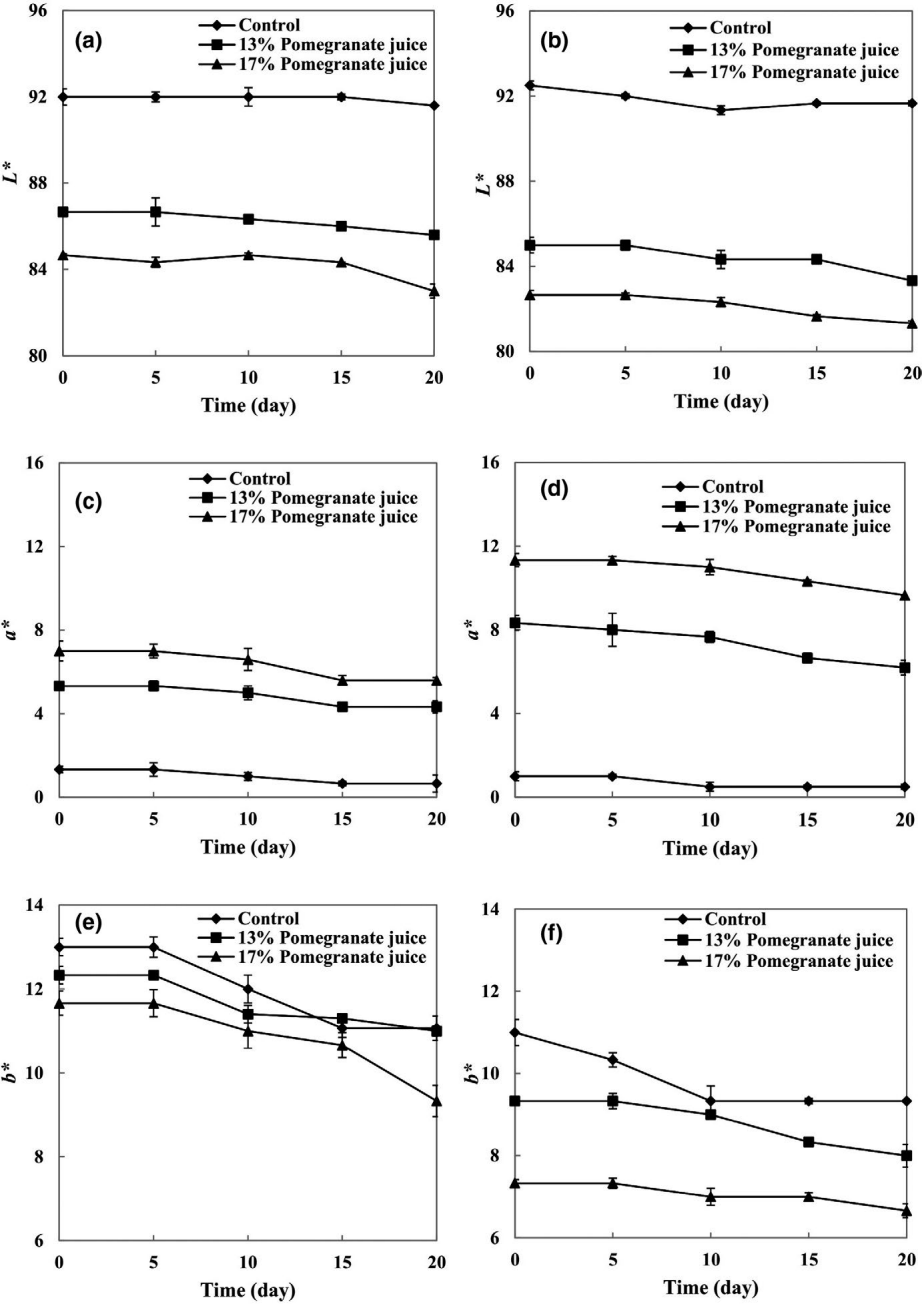
Changes in color attributes of set (a, c, e) and stirred yogurts (b, d, f) containing different concentrations of pomegranate juice during the storage period

Color attributes of yogurt samples are shown in Figure 2. As expected, the L* value decreased in response to the increase in PJ concentration (Figure 2a,b). With 17% PJ, stirred yogurts showed the lowest L* value (82), whereas the control samples of the stirred yogurts showed the highest L* value (92). This behavior can be related to the higher PJ concentration of yogurt, which increased the amount of red color in yogurt, so that its tendency decreased in being visualized as white. The type of yogurt (set or stirred) had no significant effect on the L* value. Nonetheless, at the end of the storage period, L* values of set and stirred yogurts decreased in the control (to 91 and 92, respectively), in 13% PJ‐yogurt (to 85 and 83, respectively), and in 17% PJ‐yogurt (to 83 and 81, respectively). In the course of the storage period, however, no significant difference (*p* < .05) was observed among the L* values.

According to Figure 2c,d, a* increased parallel to the increase in PJ concentration from 13% to 17%, both in the set (5–7) and stirred (8–11) yogurts. The higher PJ concentration (17%) caused the highest a* values in the yogurts, which can be related to its higher TAC. At the end of the storage period, a* values of stirred yogurts reduced from 8 to 5 by 13% PJ and from 11 to 9 by 17% PJ. Also, a* values of set yogurts decreased from 5 to 4 by 13% PJ and from 7 to 5 by 17% PJ. Karaaslan et al. (2011) reported that anthocyanins can be affected by pH, storage time, temperature, enzymes, and microbial activity, thereby explaining the fading of color or brown‐colored compounds that reduce the a* value. In our study, a decrease in a* can be related to the presence of enzymes in PJ which oxidized the phenolic compounds. Ultimately, there were no significant differences between set and stirred yogurts in terms of a* value.

By increasing the PJ concentration from 13% to 17%, b* decreased from 12 to 11 in set yogurts. Similarly, the b* value of stirred yogurts decreased from 9 to 7 by increasing the PJ concentration from 13% to 17%. During the storage period, the b* value of set yogurts decreased significantly from 12 to 11 in the 13% PJ‐yogurts and from 11 to 9 in the 17% PJ‐yogurts. Also, the b* value of stirred yogurts dropped from 9 to 8 in the 13% PJ‐yogurts and from 7 to 6 in the 17% PJ‐yogurts. Yogurt type (set or stirred) had no significant effect on the b* value.

### 3.2 Bioactive compounds

#### 3.2.1 Total phenolic content

**FIGURE 3:**
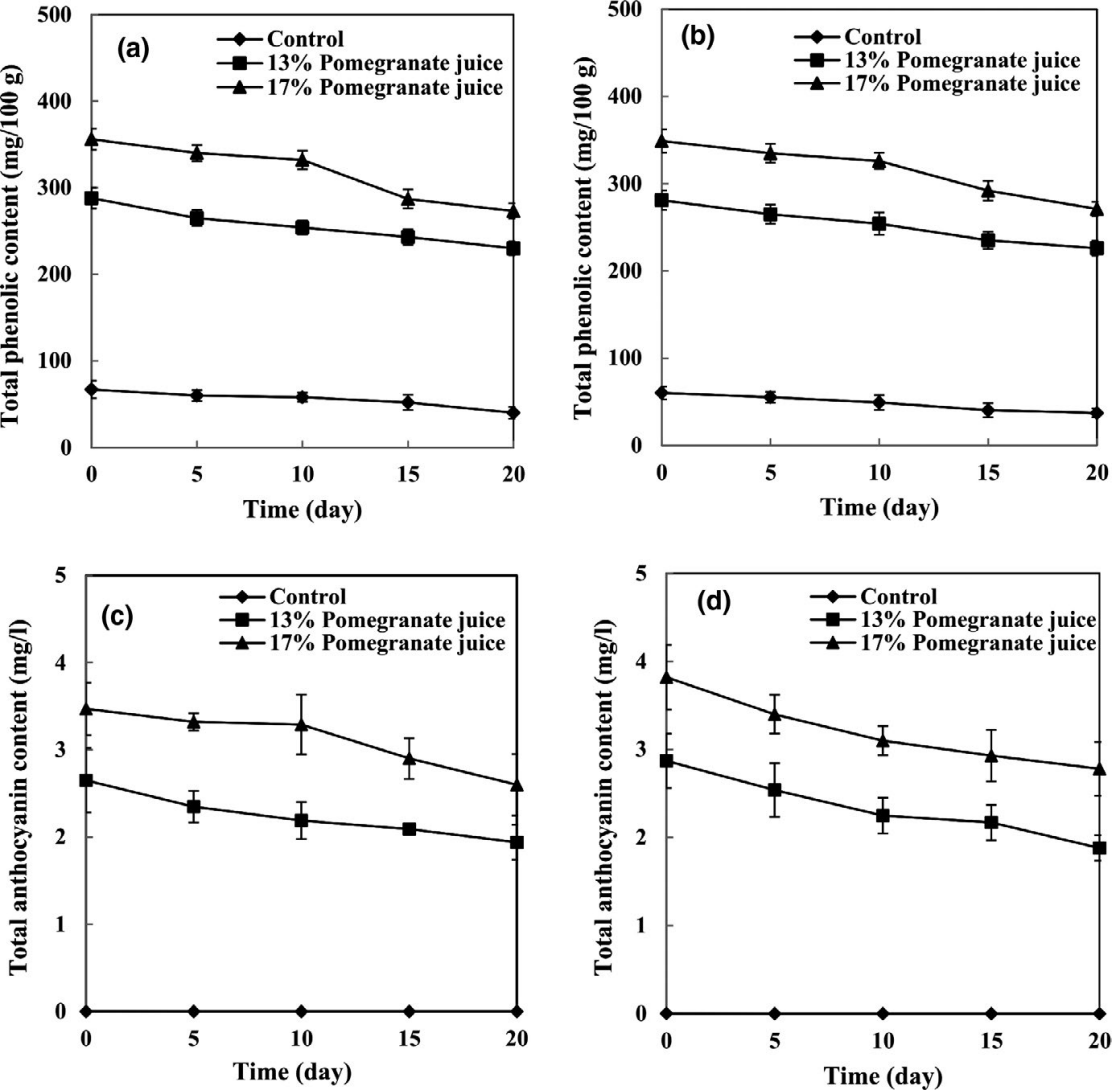
Bioactive compounds of yogurts in set (a, c) and stirred yogurts (b, d) containing different concentrations of pomegranate juice during the storage period

**FIGURE 4:**
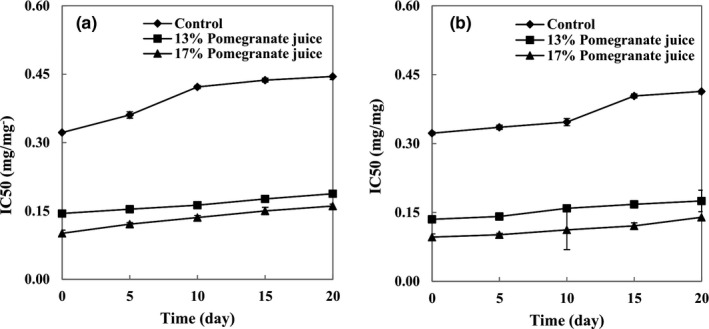
Radical scavenging activity of (a) set and (b) stirred yogurts containing different concentrations of pomegranate juice during the storage period

Total phenolic contents of set and stirred yogurts are shown in Figure 3a,b, respectively. The TPCs of set and stirred yogurts of the control group were 67 and 60 mg gallic acid/100 g at the beginning of the storage period, respectively. By increasing the PJ concentration in set yogurts from 13% to 17%, the TPC significantly increased from 288 to 356 mg gallic acid/100 g. Also, the TPC increased significantly from 281 to 349 mg gallic acid/100 g by increasing the PJ concentration similarly in stirred yogurts. This increase can be related to the presence of high amounts of polyphenol compounds (980 mg gallic acid/100 g) in PJ. In this regard, Nguyen and Hwang (2016) supplemented yogurt with 1%, 2%, and 3% aronia juice and investigated the amounts of TPC. They reported that the highest TPC (54.05 mg gallic acid/100 g) was obtained in the yogurt supplemented with the highest concentration (3%) of aronia juice, whereas the lowest TPC (16.34 mg gallic acid/100 g) was found in the control yogurt. In our study, however, measurements at the end of the storage period showed that TPC in set yogurts decreased significantly from 288 to 230 mg gallic acid/100 g and from 356 to 273 mg gallic acid/100 g by the 13% and 17% PJ, respectively. Regarding 13% and 17% PJ‐stirred‐yogurts, the TPC decreased significantly from 281 to 226 mg gallic acid/100 g and from 349 to 271 mg gallic acid/100 g at the end of the storage period, respectively. This reduction can be explained by the interactions between polyphenols and milk proteins and by the formation of insoluble complexes which consequently reduced the total amount of free polyphenols (Oliveira et al., 2015). Trigueros et al. (2014) reported a similar pattern of TPC reduction in yogurts enriched with phenolic compounds. Similarly, TPC of chocolate milk reduced due to the strong catechin‐protein interaction. Also, TPC reduction was observed when phenolic compounds of black and green tea interacted with milk proteins and pure caseins (Oliveira et al., 2015). The stability of pigments and phenols in yogurts is affected by the storage temperature, pH, phenolic content, fat, and type of bacterial culture in use. According to Figure 4a,b, the PJ concentration had positive effects on the TPC, whereas the duration of storage had negative effects. In this regard, the highest TPC was measured in the 17% PJ‐stirred‐yogurt (13.9 mg gallic acid/100 g) at the beginning of the storage period, whereas the lowest amount of TPC (4.5 mg gallic acid/100 g) was observed in the control sample at the end of the storage period. Also, the yogurt type (set or stirred) had no significant effect on TPC.

#### 3.2.2 Total anthocyanin content

Total anthocyanin contents of set and stirred yogurts are shown in Figure 3c,d, respectively. According to the obtained results, anthocyanins were not detectable in the control group of the set and stirred yogurt samples. As expected, the TAC increased (*p* < .05) by increasing the PJ concentration, confirming its direct effect on TAC. Also, TACs of the yogurts are reflected in the color properties of the samples. In agreement with our results, Jaster et al. (2018) reported that the incorporation of cryoconcentrated strawberry pulp in yogurt resulted in a product that had a TAC amount three times higher than the control. At the beginning of the storage period, the control set and stirred yogurts revealed the lowest TAC (4.5 and 1.98 mg/L, respectively). During the storage period, the TACs in yogurt samples of different PJ concentrations decreased significantly by 50%–60%. This decrease can be related to the degradation of anthocyanins, which is most likely because of the interactions between anthocyanins and milk proteins, whereby insoluble complexes are formed (Oliveira et al., 2015). However, the yogurt type (set or stirred) had no significant effect on TAC.

### 3.3 Radical scavenging activity

The IC_50_ of set and stirred yogurt samples is shown in Figure 4a,b, respectively. Stirred yogurt samples of 17% PJ showed the lowest IC_50_ value (0.096 mg/ml), indicating the highest RSA, mainly due to higher TPC and TAC values. Phenolic compounds of PJ can act as antioxidants by their ability to donate hydrogen or electrons, by terminating chain reactions, or by chelating transition metal ions. During the storage period, the IC_50_ of all yogurt samples increased significantly, as a result of anthocyanin degradation (Oliveira et al., 2015). At the end of the storage period, among the set yogurt samples, there were increases in the IC_50_ of the control, 13% PJ‐yogurt, and 17% PJ‐yogurt from 0.322 to 0.445 mg/ml, from 0.146 to 0.188 mg/ml, and from 0.101 to 0.161 mg/ml, respectively. This increasing trend was also observed in stirred yogurts, since there were increases in the IC_50_ of the control, 13% PJ‐yogurt, and 17% PJ‐yogurt from 0.323 to 0.414 mg/ml, from 0.135 to 0.175 mg/ml, and from 0.096 to 0.139 mg/ml, respectively. In addition, the yogurt type (set or stirred) had no significant effect on RSA.

### 3.4 Microbial analysis

**FIGURE 5:**
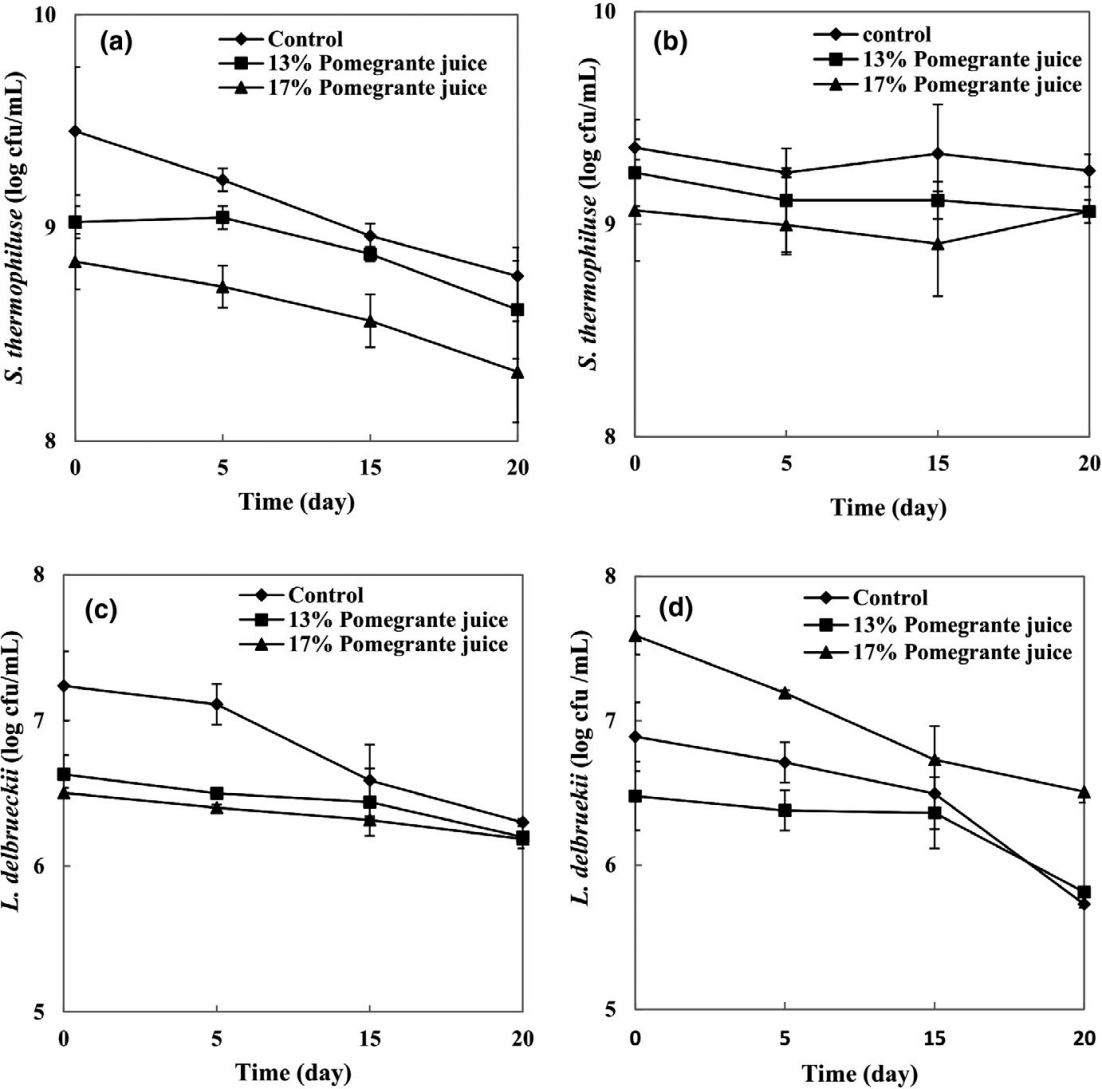
Viable counts of *Streptococcus thermophilus* and *Lactobacillus delbrueckii* subsp. *bulgaricus* during the storage period of (a, c) set and (b, d) stirred yogurts

Figure 5 shows the starter cultures and their counts in yogurt samples enriched with different concentrations of PJ. Counting the populations of *L*. *delbrueckii* in all samples revealed values higher than 6.00 log cfu/ml, although adding PJ had no significant effect (*p* < .05) on the counts of *L*. *delbrueckii*. Similarly, Cossu et al. (2009) evaluated how the addition of artichoke (*Cynara scolymus*), strawberry (*Arbutus unedo* L.), and cherry (*Prunus avium* L.) can affect microbial properties of homemade yogurts. They reported that polyphenolic extracts had no significant effect on microbial properties of yogurt samples. Also, Jaster et al. (2018) produced yogurt with different concentrations of cryoconcentrated strawberry pulp (15% and 30%) while reporting that adding cryoconcentrated strawberry cannot affect *L. delbrueckii* counts and that the count was maintained over 10^8^ cfu/ml.

**FIGURE 6:**
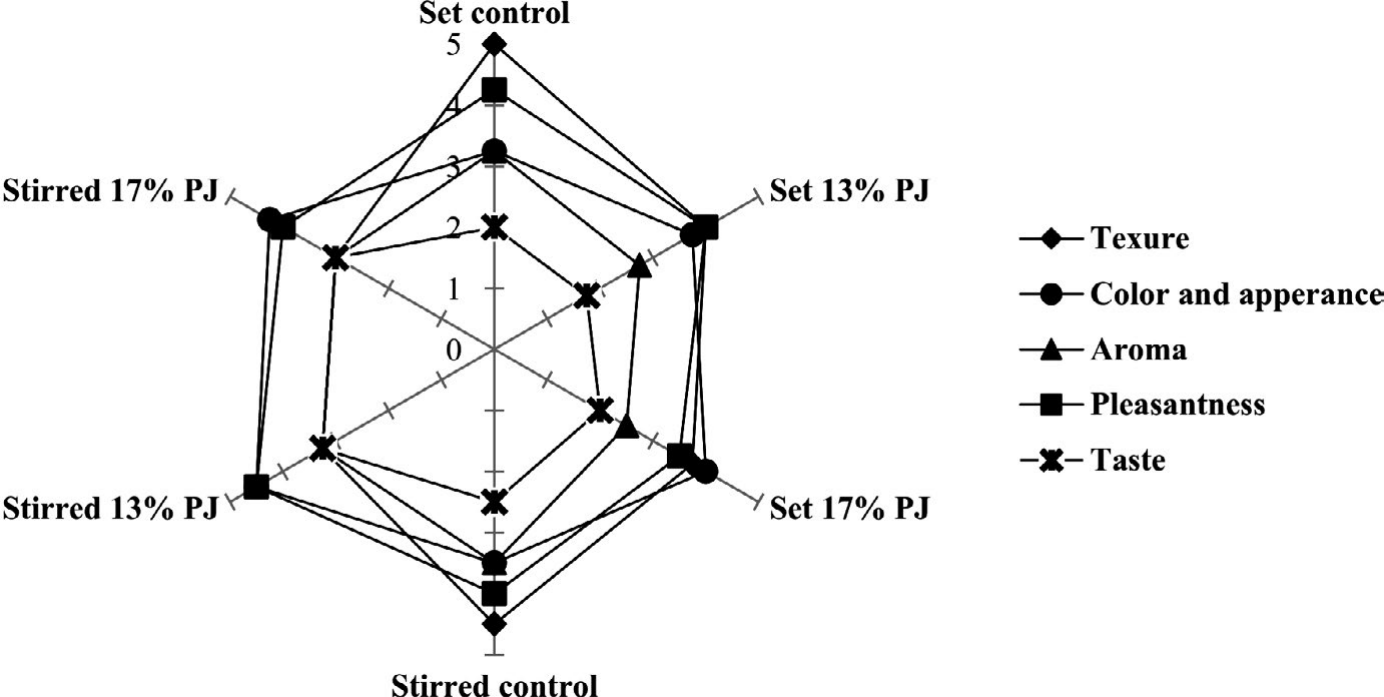
Mean score values reported by panelists (*n* = 20) for sensory properties of yogurts containing different concentrations of pomegranate juice (PJ)

During the storage period, a significant decrease (*p* < .05) was observed in *L. delbrueckii* populations. This reduction can be attributed to the accumulation of ambient lactic acid that suppressed the microbial growth. Jaster et al. (2018) observed the same results in yogurt supplemented with concentrated strawberry pulp and reported a significant reduction (*p* < .05) in *L. delbrueckii* count at the end of the storage period, as a result of an increase in acidity. According to Figure 6c,d, the yogurt type had significant effects (*p* < .05) on *L. delbrueckii* counts. Stirred yogurt hosted higher *L. delbrueckii* counts which could be seen as a result of adding PJ into the stirred yogurt (i.e., after incubation). Contents of the PJ may facilitate the increase in the *L. delbrueckii* population.

According to Figure 5a,b, increasing the PJ concentration from 13% to 17% caused a significant decrease in *S*. *thermophilus* counts, and this is probably a result of damage to bacterial cells by acidity (Vinderola et al., 2000). In all types of yogurt samples, viable counts of *S. thermophilus* were higher than those of *L*. *delbrueckii* during the storage period. This result can be attributed to the oxygen sensitivity of *L. delbrueckii*, since oxygen was introduced in the system at the moment of PJ incorporation, whereas *S. thermophilus* is known to overcome the stress caused by dissolved oxygen in the medium (Oliveira et al., 2015). The type of yogurt had a significant role in determining the *S. thermophilus* population. In fact, its count was significantly higher in stirred PJ‐yogurts (8.86–9.24 × 10^8^ cfu/ml) compared to those of set PJ‐yogurts (8.32–9.02 cfu/ml × 10^8^ cfu/ml), which can be related to the manufacturing process (i.e., PJ addition step). In general, bacterial counts in the control samples (*S. thermophilus* and *L*. *delbrueckii*) were higher than those of PJ‐yogurt samples (both 13% and 17% PJ) during the storage period. Accordingly, adding PJ had a negative impact on the starter culture count, due to the acid injury. In accordance with our findings, Vinderola et al. (2000) reported that natural fruit juices weakly inhibited *S*. *thermophilus* strains and all strains of *Lactobacilli*, except *L. casei* group strains. According to Figures 1a and 2b, it seems that the decrease in pH in the yogurt base during the manufacturing process can contribute to a lower viability of starter culture counts in the stirred and the set PJ‐yogurts.

### 3.5 Sensorial evaluation

Sensorial evaluations of yogurt samples are shown in Figure 6. Generally, PJ‐yogurts scored lower on average regarding aroma and taste by the panelists, compared to their corresponding control samples. Adding PJ imparted a sour taste that was not acceptable by some panelists. Also, lower *Lactobacillus* counts in PJ‐yogurts caused a lower level of flavors. The color scores increased as the PJ concentration increased, whereas control samples showed the lowest scores. Better color scores of PJ‐yogurts reflected higher values of TAC in the PJ which increased red pigments in the yogurt samples. The lowest texture score (3) was observed in 17% PJ stirred yogurt, possibly because of its higher syneresis. On the other hand, the highest texture score (5) was observed in the control group of set yogurt, indicating that adding PJ to yogurts had negative effects on the texture score. Regarding the pleasantness, there were no significant differences between 13% PJ and control yogurt samples. However, 13% PJ stirred yogurt sample had a higher score (4.50) than the control sample (4.25). Our findings are in agreement with Rahman et al. (2020), who claimed that the organoleptic characteristics of strawberry juice yogurt were almost as like as control yogurt. Hedonic threshold methodology (HTM) can be used as a novel method to study the acceptance of food products. de Souza et al. (2021) applied HTM to evaluate sucrose reduction in dairy products. The HTM establishes a direct relationship between stimulus intensity and sensory acceptance and does not infer that differences perceived by consumers will interfere with the sensory acceptance of the food product. So, this method is useful in test situations and provides more accurate information on sensory acceptance.

## 4 CONCLUSION

Different concentrations of PJ were added successfully to the set and stirred yogurts to improve their functional properties. Yogurt in combination with fruit juice has a functional role in the human body due to the supply of polyphenols, anthocyanins, phytonutrients, vitamins, and minerals. So, addition of PJ into the yogurt formulation led to a product with higher TPC and TAC as well as enhanced levels of antioxidant activity. However, the yogurts underwent acidification when the PJ was added to the yogurt, leading to a consequent reduction of pH, which increased yogurt syneresis. A higher syneresis of PJ‐yogurt caused lower texture quality. Adding PJ to yogurt had negative effects on starter culture counts because of the damage caused by acids. In conclusion, low concentrations of PJ (i.e., 13%) can be proposed as an excellent alternative for the purpose of producing flavored yogurt with high nutritional quality.

## CONFLICT OF INTEREST

The authors declare no conflict of interest.

## AUTHOR CONTRIBUTIONS

**Mohammad‐Taghi Golmakani:** Conceptualization (equal); Funding acquisition (equal); Project administration (equal); Resources (equal); Supervision (equal); Validation (equal); Writing‐review & editing (equal). **Mohammad Hadi Eskandari:** Conceptualization (equal); Funding acquisition (equal); Project administration (equal); Resources (equal); Supervision (equal); Validation (equal). **Somayyeh Kooshesh:** Formal analysis (equal); Methodology (equal); Software (equal). **Mahboobeh Pishan:** Data curation (equal); Formal analysis (equal); Software (equal); Writing‐original draft (equal).

## Data Availability

Data available on request from the authors.
